# Effects of specialisation on treatment and outcomes in screen-detected breast cancers in Wales: cohort study

**DOI:** 10.1038/sj.bjc.6602894

**Published:** 2005-12-06

**Authors:** P C Allgood, M O Bachmann

**Affiliations:** 1 Department of Social Medicine, University of Bristol, Bristol BS8 2PR, UK; 2 East of England Screening Quality Assurance, Compass House, Vision Park, Chivers Way, Histon, Cambridge CB4 9AD, UK; 3 School of Medicine, Health Policy and Practice, University of East Anglia, Norwich NR4 7TJ, UK

**Keywords:** specialisation, outcomes, survival, breast cancer

## Abstract

Volume–outcome relationships have been found for management of symptomatic but not for screen-detected, breast cancers. The study included 2705 patients with breast cancer detected by the Welsh breast cancer-screening programme from its inception in 1989 to 1997. Survival was tracked until 1999. Data validity was assessed for 10% of subjects. Hospitals' and surgeons' annual patient volumes were calculated as indices of specialisation. Effects of hospital and surgeon volumes on survival were estimated using Cox regression. Surgeons' and hospitals' volumes ranged from 1 to 90, and 1 to 86 patients, respectively. Patients managed by higher volume surgeons survived significantly longer (adjusted hazards ratio for a volume difference of 10 patients per year=0.90 (95% confidence intervals 0.84–0.97)). The adjusted hazard ratio for breast cancer survival was similar (0.91 (95% confidence intervals 0.82–1.00)). This association decreased over time. Patients of higher volume surgeons were significantly more likely to have axillary surgery and impalpable excision biopsies and were less likely to have mastectomy or radiotherapy. Surgeons' specialisation in management of screen-detected breast cancers was associated with longer survival, but this effect appeared to decrease over time.

Providing high-quality care to all patients with cancer was a central aim of the National Health Service (NHS) National Cancer Plan, a major part of which was to concentrate cancer care into designated cancer units and centres (The NHS Cancer Plan, 2000). There is growing international evidence that women with breast cancer receive better care when their management is concentrated in specialised centres with higher levels of expertise, skill and organisation (Basnett *et al*, 1992; Iscoe *et al*, 1994; Lee-Feldstein *et al*, 1994; Samet *et al*, 1994; Scorpiglione *et al*, 1995). There is, however, no such evidence for patients with mainly early-stage cancers detected by screening.

A Scottish study showed a significantly lower relative risk of death for patients of specialist breast surgeons compared to patients of nonspecialist breast surgeons (adjusted RR (95% confidence interval (95% CI)) 0.84 (0.75–0.94)) (Gillis and Hole, 1996). An update of this study showed that the survival advantage associated with specialist care persisted after the introduction of screening (Kingsmore *et al*, 2003). There is also evidence of a volume–outcome relationship. Two companion studies of breast cancer in Yorkshire, recently updated, provide relatively strong evidence of better survival with larger surgeon volumes (Sainsbury *et al*, 1995a, 1995b). Another study from the Thames Cancer Registry found a significant trend with increasing hospital volume of increasing access to services meeting quality of care criteria for women with breast cancer (Mikeljevic *et al*, 2003). Most of these studies concerned symptomatic breast cancers. None of them focused solely on breast cancers detected by screening. Patients with breast cancer detected by screening tend to be at earlier stages than symptomatic cancers and would tend to have higher cure rates.

The Welsh breast-screening programme, Breast Test Wales (BTW), first started screening in February 1989 when the centre in Cardiff opened serving South East Wales. October 1991 saw the opening of the centre in Llandudno and the start of screening in North Wales. This was followed shortly afterwards, in January 1992, with the opening of the Swansea centre serving West Wales.

In the early years of the screening programme, quality assurance did not extend beyond initial diagnosis and data on subsequent management were not routinely collected. The production of good quality surgical data has taken time to develop but has greatly improved in recent years. This was mainly due to the British Association of Surgical Oncology (BASO), which in the early 1990s brought together surgeons working in breast cancer screening. The BASO Breast Audit Group was set up in order to provide reliable information on treatment events for women diagnosed through the UK NHS Breast Screening Programme (NHSBSP) and to assess surgical performance. It has now become a regular part of the NHSBSP activity.

The UK programme is thus now subject to stringent quality assurance, which would be expected to have a beneficial effect on the care of women with screen-detected breast cancer. Nevertheless, two studies report wide variation between clinicians in the subsequent management of screen-detected breast cancers (Chouillet *et al*, 1994; Moritz *et al*, 1997). Suboptimal treatment of breast cancer after diagnosis could potentially compromise the effectiveness of the UK NHSBSP, but the evidence for an association between specialisation, however defined, and breast cancer can be confusing. Evidence of a higher volume effect for screen-detected breast cancer is still lacking. There was thus a need for a comparative study of the treatment and health outcomes for screen-detected breast cancer women, taking into account differences in case mix. Data held by BTW provided an excellent opportunity to study such issues in Wales. Furthermore, these data allowed for investigation of the evolution of cancer care over time, while screening and treatment policies were changing.

The main aims of this study were, therefore, to describe trends in care processes and outcomes among screen-detected breast cancer cases in Wales and to examine relationships between surgeon and hospital volumes and care processes and outcomes.

## PATIENTS AND METHODS

The study had a dynamic cohort design. Some data were recorded before and some were recorded after the start of the study. The study population comprised all women newly diagnosed by screening as having breast cancer, and managed by BTW from the start of screening in February 1989 until a last screening invitation of 31 March 1997. All patients were diagnosed by screening, and no symptomatic patients were included.

The primary data were those routinely collected by BTW and recorded on the BTW database. Sources of these data included the screening units, the pathology laboratories and surgeons. Surgeons were responsible for sending completed follow-up data to BTW.

Variables examined in this study were age at diagnosis, year of diagnosis, postal code, diagnostic investigations (nonsurgical needle biopsy, impalpable and palpable excision biopsies), the main treatment (breast conservation therapy (BCT) or mastectomy), adjuvant therapy provision (tamoxifen, radiotherapy and chemotherapy provision), prognostic indicators for invasive tumours (size, histological grade and spread), recurrences and deaths (all cause and breast cancer specific).

In all, 15% of women (417 of 2705) had no follow-up visits recorded on the BTW database suggesting incomplete reporting. For these women, data were collected from hospital records by PA. There was also concern by BTW that some adjuvant therapy details were not being fully reported. In order to validate the data on the BTW database, 10% of all women were randomly sampled using computer-generated random numbers, and their hospital records were examined (by PA). The adjuvant therapy details for this random sample were also obtained from the women's hospital records at the same time as the missing follow-up details. Once the information had been collected, the data were anonymised.

In cases where there was no record of a woman having received a treatment, for example, tamoxifen, chemotherapy or radiotherapy, it was assumed that she did not receive it.

Each woman was allotted a surgeon volume and a hospital volume, which were the annual patient volumes for their main surgeon and main hospital. Patient volumes were defined as the number of newly diagnosed women with screen-detected breast cancer that was managed by each surgeon or hospital per year. Each patient's main surgeon was defined as the surgeon who carried out the most radical operation, as this was the procedure most likely to influence survival. Similarly, the hospital where the most radical surgery was carried out was classed as the main treatment location. Townsend deprivation scores were derived from each patient's postal code, and linked to enumeration district data from the 1991 census (Morris and Carstairs, 1991).

Patient survival data were tracked with the NHS Central Register and updated quarterly. The register was last updated for this study on 31 January 1999. Death details specified dates and primary and contributory causes of death. Women still alive at this time were defined as censored in the survival analysis.

Conventional statistical methods were used for summary descriptions of variables (Hamilton, 1998; Altman, 1991). A 5% significance level was used. Statistical analysis primarily emphasised estimation of differences and ratios, with 95% CI. Proportions were compared using Pearson's *χ*^2^ and exact tests. Trends in proportions across ordinal categorical variables were tested using Spearman's rank correlation or Cuzick's test for trend (Cuzick, 1985).

Multiple logistic regression was used to examine the independent relationships between dichotomous outcome variables and several explanatory variables. Cox's proportional hazards model was used for multivariable survival analysis. Cox regressions were stratified by variables that had nonproportional hazards (Parmar and Machin, 1995). Explanatory variables were entered into the multivariable models if they were thought *a priori* to be potentially causally related to or potentially confounding factors for the outcome of interest. All putative causes preceded their respective outcomes chronologically. Surgeon and hospital volumes were included in all models as they were the main explanatory variables of interest. Adjusted odds ratio and hazards' ratio (HR) were estimated by using surgeon and hospital volumes as continuous variables. They were also transformed into ordinal categorical variables with low, medium and high volume categories. Cut-points for choosing high, medium or low volumes were arbitrary and were the same for both. In secondary survival analyses, different arbitrary volume cut-points for surgeon volume were also tested.

For the adjuvant therapy and survival models, analyses were firstly carried out on all tumours, that is, both invasive and *in situ*. The analyses were then repeated for invasive tumours only, so as to be able to adjust for the prognostic indicators of size, histological grade and nodal involvement, which are not comparable to *in situ* tumours.

The principal unit of analysis was a patient and the primary analysis assumed independence between women. The potentially clustered nature of the data necessitated adjustment for intrasurgeon and intrahospital correlation in the regression analysis. Adjusted standard errors and 95% CIs were estimated using Stata's ‘cluster’ option for regression analyses (STATA, 1999).

## RESULTS

### Case-mix and surgeon and hospital volumes

A total of 2705 women diagnosed with primary breast cancer were identified. They were managed by 25 surgeons in 19 hospitals. Table 1 shows patients' characteristics for different surgeon volumes. Most of the women had early-stage cancers. Although 79% were invasive, 76% had no involved nodes, 75% of tumours were less than 19 mm in diameter and 55% had a ‘good’ Nottingham Prognostic Index (Table 2).

Prognostic characteristics of women managed by high, medium and low-volume surgeons or hospitals did not differ significantly. Women of lower volume surgeons, however, tended to live in more affluent areas. Women of lower volume surgeons were also more likely to have missing tumour grades, missing Nottingham Prognostic Index and more affected nodes, but these differences were not significant.

The median annual number of patients seen by each surgeon and each hospital decreased significantly over time (*P*<0.01). Table 3 shows median surgeon and hospital volumes and ranges for different time periods. The proportion of women managed by high or medium volume surgeons/hospitals, however, remained over 95% over time. Only 3% of women were managed by surgeons, with fewer than 10 cases per year, and only 4% were managed in hospitals, with fewer than 10 cases per year. Surgeon volume and hospital volume were significantly associated with each other (*P*<0.001).

### Diagnostic and therapeutic procedures

Most women had a nonsurgical needle biopsy (1784 of 2705), over a quarter had an impalpable excision biopsy and fewer than 5% had a palpable excision biopsy. In all, 72 women had no surgical procedure after diagnosis. Of the 2663 women who did have therapeutic surgery, nearly half of them had a mastectomy. Approximately half of all 2705 women received tamoxifen, a third received radiotherapy and 3% received chemotherapy.

In the multiple logistic models (Table 4), surgeon volume was independently associated with having a mastectomy, axillary surgery and being given radiotherapy. With every increase of 10 women per surgeon per year, there was a 6% lower probability of a woman having a mastectomy rather than BCT, a 22% higher probability of having axillary surgery and a 10% lower probability of being given radiotherapy. Surgeon volume was not independently associated with having a needle biopsy, tamoxifen or chemotherapy.

Hospital volume was independently associated with having a mastectomy, radiotherapy or chemotherapy. With every increase of 10 women per hospital, there was a 5% higher probability of having a mastectomy rather than BCT, a 7% higher probability of having radiotherapy and a 16% lower probability of having chemotherapy.

### Trends over time

Biopsy procedures changed significantly over the study period. The proportion of women having a diagnostic needle biopsy increased from 44% in 1989–1990 to 75% in 1996–1997 (Cuzick's test for trend, *z*=6.10, *P*<0.01). Consequently, the proportion of women having an impalpable excision biopsy decreased (Cuzick's test for trend, *z*=−3.17, *P*<0.01). Palpable excision biopsies also decreased significantly over time (Cuzick's test for trend, *z*=−5.08, *P*<0.01). Mastectomy rates and adjuvant therapy also changed significantly over time. The proportion of women having a mastectomy rather than BCT decreased from 56 to 42% (Cuzick's test for trend, *z*=−6.64, *P*<0.01) as did the proportion of women having axillary surgery (Cuzick's test for trend, *z*=−4.9, *P*<0.01). Radiotherapy and tamoxifen provision increased significantly over time (Cuzick's test for trend, *z*=7.8, *P*<0.01, and *z*=3.8, *P*=0.01, respectively).

### Survival time

**Figure 1:**
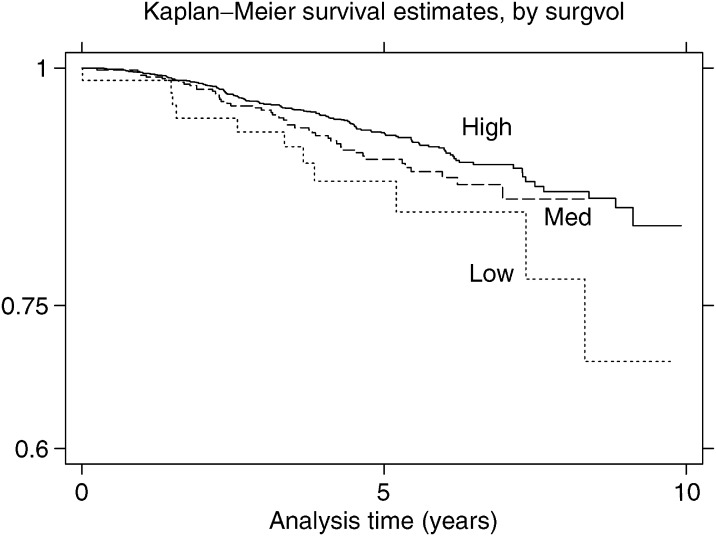
Kaplan–Meier survival curve by surgeon volume.

During the period of follow-up, 203 of the 2705 (7.4%) women died, of whom 194 had complete data for survival analysis. A total of 120 deaths (4.4% of 2705) were attributed to breast cancer and 83 to other causes. Survival was worse for older women, women with bilateral tumours, invasive tumours, or women having a mastectomy or receiving chemotherapy (Table 5). For all-cause survival, higher surgical volumes were independently associated with longer survival, but higher hospital volumes were not. Figure 1 shows unadjusted survival results for all-cause survival by surgeon volume from which clear differences can be seen.

For breast cancer survival, women had a nonsignificantly better survival if managed by higher-volume surgeons compared with lower-volume surgeons. Among the 2121 women with invasive tumours, 181 (8.5%) died, 113 of 2121 (5.3%) were from breast cancer. In this subgroup, tumour size, histological grade and number of involved nodes (which were only obtained for invasive tumours) were shown to be strong independent predictors of both all-cause and breast cancer survival. Women with large tumours, tumours with node involvement and tumours of a more severe histological grade had worse survival (*P*-values=0.002, <0.001, <0.001, respectively, for both all-cause and breast cancer). Hazard ratios for surgeon and hospital volumes were not substantially different for the invasive tumour subgroup than for all patients (HR (95% CI): 0.990 (0.982–0.997, *P*=0.009, and 1.003 (0.996–1.010), *P*=0.38, respectively, for all-cause survival). The HR for surgeon volume for breast cancer survival for women with invasive tumours was similar to that from all cause (HR (95% CI): 0.991 (0.981–1.001, *P*=0.08)), although the difference was marginally nonsignificant owing to fewer events. Residence in a more or less deprived area did not influence survival in any of the analyses (*P*=0.83).

When time trends were examined, the association between surgeon volume and survival decreased over time. The HR associated with a difference in surgeon volume of 10 women was 0.86 (95% CI: 0.78–0.94) in women diagnosed during 1988–1993, 0.97 (95% CI: 0.93–1.13) in women diagnosed during 1993–1995 and 0.99 (95% CI: 0.97–1.23) in women diagnosed during 1995–1997. However, this trend was not statistically significant (*P*-value for adding period × volume interaction term to the model=0.57). These findings were robust despite extensive sensitivity analyses using alternative methods of data analysis, including examining survival until death from breast cancer, different periods of follow-up, different ways of calculating surgeon and hospital volumes, different ways of dealing with missing prognostic and adjuvant therapy data, and adjustment for clustering of outcomes within surgeons and hospitals.

### Completeness of BTW data

In all, 15% (417 of 2705) of women had missing follow-up data recorded at BTW. Results of the regression analysis showed that, overall, surgeon volume was the strongest predictor of whether data for follow-up visits were recorded or not (*P*<0.001). As surgeon volume increased, information for follow-up visits was more likely to be recorded. Hospital volume and period of diagnosis also significantly influenced the recording of follow-up information (*P*<0.001 for both). Data were less likely to be missing as hospital volume increased, or if a woman was diagnosed in the screening period 1993–1995 compared to the earliest diagnostic period, 1988–1993.

Validation of the 10% random sample estimated the sensitivity of BTW data, defined as the proportion of patients for whom an item recorded in hospital files was also recorded by BTW. This showed that, for radiotherapy provision, sensitivity ranged from 58% for low-volume surgeons to 80% for high-volume surgeons. For tamoxifen provision, sensitivity was 62% for high-volume surgeons compared with 43% for low-volume surgeons. Sensitivities for both radiotherapy and tamoxifen provision differed significantly between higher- and lower-volume surgeons (*P*=0.06 and 0.001, respectively).

In the survival models, sensitivity analyses were conducted to assess the robustness of results to assumptions about the validity of information on radiotherapy and tamoxifen provision. This entailed assuming, first, that those with no information had received these treatments and, second, that they had not. Changing these assumptions did not substantially change the results.

## DISCUSSION

This study shows that specialisation, as indicated by the annual caseload of surgeons and to a lesser extent hospitals, influenced the choice of surgical treatment, adjuvant therapy provision and survival time during the first decade of the breast-screening programme. It therefore supports the specialisation of early breast cancer care. Higher-volume surgeons were more likely to provide more effective treatment (axillary surgery), were less likely to provide the most traumatic treatments (mastectomy) and obtained the longest survival, independently of prognostic features. While the greatest problems with management and outcomes appeared to be at the lowest end of the surgeon volume spectrum, there were no clear volume thresholds.

For many of the variables measured, including treatment procedures and survival, there were consistent and significant trends of better clinical practice associated with increasing surgeon volume. This was not so evident with increasing hospital volume, suggesting that a woman's breast cancer management was influenced more by the experience of the surgeon in whose care she was than by the experience of the hospital in which she was treated. This may explain the surprising association between hospital volume and main treatment procedure despite the positive correlation between surgeon and hospital volumes; one would expect that the choice of treatment would depend primarily on the surgeon rather than on the hospital. However, over 25% of lower volume surgeons also worked in higher volume hospitals, suggesting that these hospitals needed to address the problem of unspecialised surgeons within them.

Volume–outcome relationships for breast cancer survival were very similar to those for all-cause survival, although they were not statistically significant. Our figures were very similar to those in the Yorkshire study where an HR (95% CI) associated with a surgeon who managed 30 or more new cases of invasive breast cancer per year compared to one who managed 10 or less was 0.85 (0.77–0.93) (Sainsbury *et al*, 1995a). In our study, a difference of 20 women per surgeon per year in the model for all tumours gave an HR (95% CI) of 0.85 (0.74–0.98) for all-cause mortality and 0.85 (0.71–1.02) for breast cancer mortality. For invasive tumours only, for a difference of 20 women per year, we found HRs of 0.82 (0.71–0.96) for all-cause mortality and 0.82 (0.67–1.02) for breast cancer mortality. Although the Yorkshire study involved many more patients than our study and could therefore generalise with more confidence than ours, it does suggest that the main findings of the Yorkshire study may be generalised to women with earlier, screen-detected, cancers. Unlike the Yorkshire study, this study found no clear threshold for effects of surgeon volumes on survival.

Clinical management improved over time, varied between the three centres and appeared to be most in keeping with practice guidelines among women of higher volume surgeons. Lower-volume surgeons tended to perform more invasive surgery and were less likely to follow guidelines regarding axillary surgery and adjuvant therapy provision. Provision of tamoxifen and chemotherapy were, however, not associated with surgeon volume (HR: 1.003 (0.998–1.008), *P*=0.236, and 1.007 (0.993–1.21), *P*=0.327, respectively). Results suggested that older and less affluent women were not discriminated against with regard to specialist care. In this study, social deprivation did not appear to be an important prognostic factor in any of the Cox analyses. This has also been found by Haybittle *et al* (1997).

This study is original in that it measures the relationship between specialisation, as estimated by surgeon and hospital caseload, and outcomes in the breast-screening population of an entire NHS region. Other key strengths of this study include the large sample size, the prospective cohort design and the inclusion of a wide range of clinical data, which allowed for a thorough analysis controlling for these variables. All cases were tracked for survival. The use of surgeon and hospital volumes proved a robust and objective measure of specialisation.

In calculating surgeon and hospital volumes, this study could not take into account surgeons' experience in management of symptomatic nonscreening-detected breast cancer, which could plausibly affect their skills in managing screen-detected cancers. Total breast cancer volumes, which may thus be a more relevant index of specialisation, would be greater than the volumes for screen-detected breast cancers used in this study, but would be unlikely to affect our main findings if volumes of symptomatic and screen-detected patients were highly correlated. Surgeon's total breast cancer volumes could be estimated by analysis of cancer registry data and hospital episode statistics, but these data are not held by BTW and so were beyond the scope of this study. This deserves further investigation, however, and we are currently investigating the value of linked cancer registry data and hospital episode statistics to assess volume–outcome relationships in another region.

If, as is common practice, patients with impalpable breast cancers were selectively referred to a few designated surgeons, while all other patients were referred to local breast surgeons, then it is possible that patients of lower-volume surgeons, as defined by us, could tend to have less advanced disease, with better outcomes. However, this does not appear to be the case in this study. Patients of lower-volume surgeons did not have smaller or less invasive tumours (Table 2). Impalpable tumours were not recorded as such. And patients of lower volume surgeons tended to have poorer, not better, outcomes. So this kind of selective referral, if present, would tend to dilute the volume–outcome associations we have shown here, rather than spuriously to produce associations when none existed.

This study's observational design shows what actually happens in practice when screening and treatment are applied to large populations. However, observational studies are susceptible to confounding. Differences in disease severity between women of higher- and lower-volume surgeons and hospitals are of particular concern. For this reason, efforts were made to obtain valid data on baseline prognostic factors and treatments. These data were generally more complete than most cancer registries would allow. The study carefully measured and controlled for the main prognostic factors. Secondary analyses were carried out which supported the main results, suggesting that confounding was not a major problem in this study.

Although breast cancer care is multidisciplinary, embracing many health professions and specialities, there still appeared to be a small proportion of surgeons not actively participating in best protocol. Although approximately 97% of women in this study were treated by relatively high-volume surgeons, 3% were managed by surgeons treating on average less than nine women per year and these women had worse survival. Surgeons (40%) in the low-volume category managed an average of only one screening case per year. This is a cause for concern and needs to be investigated further. Surgeon volume is now addressed each year in the BASO audit. Breast cancer should be cared for by surgeons with a specialist interest in breast cancer and a high breast cancer caseload.

Follow-up audit is essential for continuing quality improvement. It would also be valuable to continue to monitor survival over longer periods, given the relatively low mortality rates in the earlier years after screening. This study shows the value of collection and analysis of case mix and outcome data in addition to examination of care processes alone.

## Figures and Tables

**Table 1:** Surgeon volume and patient demographic characteristics, follow-up duration and survival

**Surgeon volume^a^ (*N*)**	**High (6) (⩾50)**	**Medium (4) (11–49)**	**Low (15) (⩽10)**	**Total (25)**	**Test, *P*-value**
*Age at diagnosis (years)*					Spearman's rank
*N* (%)	2092	536	76	2704	correlation
Mean (s.d.)	58.6 (5.6)	58.7 (5.7)	58.5 (4.9)	58.5 (5.6)	*P*=0.48
Range	41.3–81.0	49.2–84.9	41.4–72.9	41.3–84.9	
					
*Townsend deprivation score*					ANOVA
1st quartile (most affluent)	482 (23)	167 (31)	27 (36)	676 (25)	*P*=0.001
2nd quartile	553 (26)	106 (20)	11 (14)	670 (25)	
3rd quartile	511 (24)	142 (26)	21 (28)	674 (25)	
4th quartile	539 (26)	118 (22)	17 (22)	674 (25)	
Missing^b^	7 (0.3)	3 (0.6)	0	10 (0.4)	
					
*Follow-up (years)*					Spearman's rank
*N* (%)	2086	534	75	2696	correlation
Median (IQR)	3.0 (1.9–4.2)	3.0 (1.5–4.2)	3.1 (2.0–4.9)	3.0 (1.8–4.2)	*P*<0.001
Missing^b^	6 (0.29)	2 (0.37)	1 (1.3)	9 (0.33)	
					
*Recurrence*					Wilcoxin's rank sum
*N* (%)	87 (4.2)	26 (4.9)	5 (6.9)	118 (4.4)	*P*=0.69
					
*All-cause mortality*					Wilcoxin's rank sum
*N* (%)	146 (7.1)	46 (8.6)	11 (4.5)	203 (7.5)	*P*=0.76
					
*Breast cancer mortality*					Wilcoxin's rank sum
*N* (%)	86 (4.1)	28 (5.2)	6 (7.9)	120 (4.4)	*P*=0.9996

ANOVA=analysis of variance; IQR=interquartile range.

All tumours *N* (%): 2704 (100), one patient with missing surgeon ID.

a Surgeon volume analysed as a continuous variable.

b Missing values not used in analyses.

**Table 2:** Surgeon volume and treatment and histological prognostic factors

	**High (6) (⩾50)**	**Medium (4) (11–49)**	**Low (15) (⩽10)**	**Total (25)**	**Test, *P*-value**
*Surgeon volume*^a^, *N*					
Therapeutic procedures, *N* (%)					Cusick's test for trend
BCT	1133 (54)	299 (56)	29 (38)	1461 (54)	*z*=1.15, *P*=0.25
Radiotherapy	724 (35)	169 (32)	615 (20)	908 (34)	*z*=−2.6, *P*=0.014
Tamoxifen	1129 (54)	211 (39)	21 (28)	1361 (50)	*z*=−7.23, *P*<0.01
Chemotherapy	74 (3.5)	9 (1.7)	4 (5.3)	88 (3.3)	*z*=−1.13, *P*=0.26
					
Invasive status, *N* (%)					Wilcoxin's rank sum
Invasive tumours	1636 (78)	422 (79)	63 (83)	2121 (78)	*P*=0.97
*In situ* tumours	448 (21)	112 (21)	12 (16)	572 (21)	
Missing^b^	8 (0.38)	2 (0.37)	1 (1.32)	11 (0.41)	
All tumours	2092	536	76	2704	
					
*Invasive tumours only*^c^, *N* (%): *2121 (100)*					
Malignant nodes^a^					Spearman's rank
None	1221 (75)	312 (74)	37 (59)	1570 (74)	correlation
Few (1–4)	299 (18)	79 (19)	18 (29)	396 (19)	*P*=0.59
Medium (5–9)	60 (3.7)	14 (3.3)	3 (4.8)	77 (3.6)	
Lots (⩾10)	18 (1.1)	9 (2.1)	0	27 (1.3)	
Missing^b^	38 (2.3)	8 (1.9)	5 (7.9)	51 (2.4)	
					
Size (mm)^a^, *N*	1620	417	60	2097	Spearman's rank
Median (IQR)	13 (9–18)	13 (9–19)	15 (10.5–22)	13 (9–18)	correlation
Range	1–99	1–99	2–60	1–99	*P*=0.53
Missing^b^	16 (0.8)	5 (0.9)	3 (3.9)	24 (0.9)	
					
					
Grade					
1	528 (32)	115 (27)	10 (16)	653 (31)	Cusick's test
2	711 (43)	181 (43)	27 (43)	919 (43)	for trend
3	224 (14)	55 (13)	9 (14)	288 (14)	*P*=0.85
Missing^b^	173 (11)	71 (17)	17 (27)	261 (12)	
					
NPI^a^					Spearman rank
Good	936 (57)	220 (52)	19 (30)	1175 (55)	Correlation
Moderate	391 (24)	106 (25)	19 (30)	516 (24)	*P*=0.94
Poor	87 (5.3)	17 (4.0)	4 (6.4)	108 (5.1)	
Missing^b^	222 (14)	79 (19)	21 (33)	322 (15)	

BCT=breast conservation therapy; IQR=interquartile range.

All tumours, *N* (%): 2704 (100), one patient with missing surgeon ID.

a Surgeon volume, malignant nodes, size and Nottingham Prognostic Index analysed as continuous variables.

b Missing values not included in analyses.

c These data not available for noninvasive tumours.

**Table 3:** Surgeon and hospital patient volumes for different time-periods

**Period**	**1988–1993**	**1993–1995**	**1995–1997**	**Total**	**Cusick's test for trend^a^**
Surgeon volume^b^	67 (47–90) 1–90	58 (51–67) 1–90	58 (51–67) 1–90	65 (51–90) 1–90	*z*=−14.72, *P*<0.01
Hospital volume^b^	83 (64–86) 1–86	68 (44–83) 1–86	64 (34–83) 1–86	68 (44–83) 1–86	*z*=−18.66, *P*<0.01

a Surgeon and hospital volumes, and year analysed as continuous variables.

b Median (interquartile range) range.

**Table 4:** Effect of a difference in surgeon^a^ or hospital volume^a^ of 10 women on the provision of diagnostic and therapeutic procedures: logistic regression models^a^

**Outcome variable**	**% received**	**Explanatory variable**	**Adjusted odds ratio**	**95% CI**	***P*-value**
Needle biopsy^b^	66.0	Surgeon volume	1.00	0.96–1.05	0.84
Mastectomy^c^	47.2	Surgeon volume	0.94	0.90–0.98	0.007
		Hospital volume	1.05	1.01–1.08	0.011
Axillary surgery^d^	93.5	Surgeon volume	1.22	1.10–1.34	<0.001
Radiotherapy^e^	40.3	Surgeon volume	0.91	0.85–0.98	0.015
		Hospital volume	1.07	1.01–1.14	0.024
Tamoxifen^f^	56.3	Surgeon volume	1.04	0.98–1.10	0.20
Chemotherapy^g^	3.8	Hospital volume	0.85	0.76–0.95	0.006

CI=confidence interval.

a Surgeon and hospital volume analysed as continuous variables and included in all models.

b Also adjusted for age, centre and period.

c Also adjusted for age, centre, period, bilateral tumours, invasive status and the Townsend Deprivation Score.

d Also adjusted for centre, period, therapeutic procedure and bilateral tumours.

e Also adjusted for centre, period, therapeutic procedure, tamoxifen, size, grade and nodal status.

f Also adjusted for centre, period, therapeutic procedure, age, size, grade and nodal status.

g Also adjusted for age, size, grade and nodal status.

**Table 5:** Significant^a^ predictors of survival time until death from any cause: Cox's proportional-hazards model

	**Crude HR**	**95% CI**	**Adjusted HR^b^**	**95%** **CI**	**Adjusted *P*-value**
*Explanatory variable*					
Surgeon volume^d^	0.91	0.85-0.097	0.90	0.84–0.97	0.008
Hospital volume^d^	1.00	0.94–1.06	1.04	0.98–1.12	0.213
Age at diagnosis (per year)	1.04	1.01–1.06	1.04	1.02–1.07	0.001
Invasive *vs* *in situ*	2.49	1.55–3.99	2.33	1.39–3.90	0.0003
Bilateral *vs* unilateral	3.35	1.82–6.15	2.93	1.54–5.87	0.005
Mastectomy *vs* BCT	2.09	1.55–2.82	1.79	1.32–2.44	0.0001
					
*Invasive tumours, N*=*2121 (100*%)					
Surgeon volume^d^	0.91	0.85–0.97	0.91	0.84–0.98	0.011
Hospital volume^d^	1.00	0.94–1.06	1.03	0.84–0.97	0.009
Age at diagnosis (per year)	1.04	1.10–1.06	1.03	0.96–1.11	0.38
Bilateral *vs* unilateral	3.35	1.82–6.15	2.31	1.00–1.06	0.05
Mastectomy *vs* BCT	2.09	1.55–2.82	1.46	1.15–4.63	0.02
Chemotherapy *vs* none	6.40	4.26–9.63	2.35	1.04–2.05	0.03
Size	1.03	1.02–1.04	1.02	1.40–3.94	0.001
Grade 1 vs					
grade 2	1.52	1.00–2.33	1.23	1.01–1.03	0.002
grade 3	5.09	3.33–7.78	3.00	0.80–1.90	<0.0001
Missing grade 3	1.34	0.76–2.37	0.94	1.91–4.72	<0.0001
					
No malignant nodes *vs*					
1–4	2.31	1.63–3.28	1.65	0.52–1.71	
5–9	7.08	4.50–11.13	3.47	1.14–2.38	
⩾10	12.48	7.08–21.99	4.26	2.06–5.86	
				2.19–8.29	

CI=confidence interval; HR=hazards ratio.

All tumours, *N*=2705 (100%).

a Hospital volume not significant, but included because it is one of the main explanatory variables of interest.

b Adjusted for all the other variables in the model except for size, grade and malignant nodes, which were only relevant to the invasive tumours model.

d For a difference in mean volume of 10 patients per year.
